# Nonradiographic axial spondyloarthritis: clinical and therapeutic relevance

**DOI:** 10.1186/s13075-017-1493-8

**Published:** 2017-12-22

**Authors:** Nilasha Ghosh, Eric M. Ruderman

**Affiliations:** Northwestern University Feinberg School of Medicine, 675 North St. Clair, Suite 14-100, Chicago, IL 60611 USA

**Keywords:** Axial spondyloarthritis, Nonradiographic axial spondyloarthritis, Disease classification, Therapy, Biologic therapy

## Abstract

Current classification criteria for axial spondyloarthritis (axSpA) provide for the inclusion of patients with a wide range of presentations and manifestations. While not considered a formal subclassification, patients are often divided into radiographic or nonradiographic axSpA based on the presence or absence of radiographic sacroiliitis. This review will focus on nonradiographic axSpA and will discuss clinical manifestations of disease that distinguish, or in many cases do not distinguish, this entity from other individuals with axSpA. This review will also cover treatment paradigms for nonradiographic axSpA, particularly the use of biologic therapies, where current data suggest that nonradiographic disease should be managed largely the same as radiographic disease, or classical ankylosing spondylitis.

## Background

Spondyloarthritis (SpA) refers to a family of chronic rheumatic disorders of joint inflammation, including ankylosing spondylitis (AS), psoriatic arthritis, reactive arthritis, and arthritis related to inflammatory bowel disease [1, 2]. Axial spondyloarthritis (axSpA) refers to predominant inflammation in the spine and/or sacroiliac joints and is characterized clinically by inflammatory back pain and stiffness that may be improved with exercise [3]. Sacroiliac changes on conventional radiographs have long been a key element of the classification of AS, now considered a subset of axSpA [4]. With this in mind, some have suggested dividing axSpA into radiographic (r-axSpA) or nonradiographic (nr-axSpA) forms depending on the presence or absence of radiographic changes at the sacroiliac joints; under this schema, patients traditionally classified as AS would be considered r-axSpA. This review will discuss various aspects of axial spondyloarthritis, with a strong focus on nr-axSpA and its current classification in the field of rheumatology. We will review epidemiology, prognosis, current treatments, and potential future therapies.

## Search strategy

Information for the review has been drawn from manuscripts in the PubMed database published within the last 10 years. Search terms included “axial spondyloarthritis,” “non-radiographic axial spondyloarthritis,” “criteria AND axial spondyloarthritis,” “inflammatory spinal disease,” “[TNF inhibitors OR treatment] of axial spondyloarthritis,” “biomarkers AND axial spondyloarthritis,” “[predictors OR progression] of axial spondyloarthritis,” “fibromyalgia AND axial spondyloarthritis,” “therapy + axial spondyloarthritis” and “monitoring tools AND disease activity.”

## History of axSpA

AxSpA represents a spectrum of inflammatory conditions that primarily affect the axial skeleton and sacroiliac joints. AS is the prototypic disorder, with recognition as a distinct entity in the early 1900s [5]. Among the earliest criteria established for the diagnosis of AS was the modified New York (mNY) criteria, which combined clinical and conventional radiographic findings. The mNY criteria became the most widely used for clinical studies and treatment trials for many years until it was recognized that identifying sacroiliitis on plain film was a late finding in the disease course of many patients [3, 5]. In the 1990s, the Amor and the European Spondyloarthropathy Study Group (ESSG) criteria were established in an effort to capture a wider spectrum of spondyloarthropathies in the hopes of identifying patients in earlier stages of disease [6, 7]. The Amor criteria involved scoring several clinical, radiographic, and treatment response variables to indicate the presence or absence of SpA, whereas the ESSG criteria required the presence of inflammatory spinal pain or synovitis with one additional minor criterion in order to establish the diagnosis of SpA [6]. Because these criteria were designed to capture the entire spectrum of spondyloarthritis, they were too general to specifically identify early axial disease [7]. The next step, therefore, was to build upon the Amor and ESSG criteria to establish the Assessment of SpondyloArthritis International Society (ASAS) criteria [5] with the aim of identifying patients with early axial disease that is not evident on conventional radiographs.

## ASAS criteria

**Fig. 1:**
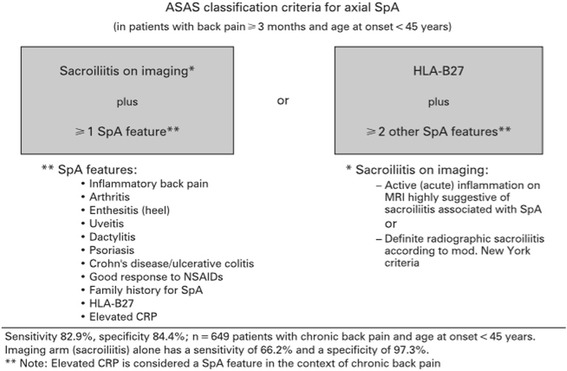
ASAS classification criteria for axial spondyloarthritis. Published with permission from [9]. *ASAS* Assessment of SpondyloArthritis International Society, *CRP* C-reactive protein, *HLA* human leukocyte antigen, *MRI* magnetic resonance imaging, *NSAIDs* nonsteroidal anti-inflammatory drugs, *SpA* spondyloarthritis

In 2009, a study of 71 patients without definite sacroiliitis by the mNY criteria was reviewed by a panel of twenty experts to determine the utility of magnetic resonance imaging (MRI) as an appropriate diagnostic tool in the recognition of early axial disease [8, 9], since bone marrow edema on MRI represents the early stages of inflammation [6]. Based on the patients’ histories, data, and imaging, a list of characteristics associated with axSpA was established, thus forming the ASAS criteria for axSpA [5, 8, 9] (Fig. 1). These criteria provide two routes through which the diagnosis can be made: either through a clinical arm (positive testing for human leukocyte antigen (HLA)-B27 plus two additional characteristic features of SpA) or a radiographic/imaging arm (radiographic sacroiliitis or active inflammation of the sacroiliac joints on MRI, plus one other characteristic feature of SpA) [8, 9]. Using MRI as a tool for the diagnosis of axSpA as the basis of the imaging arm criteria yielded high specificity (97.3%); however, sensitivity was compromised at 66.2%, thus missing about a third of the patients with axSpA [5, 8, 9]. The clinical arm alone had a sensitivity and specificity of 83.3% and 84.9%, respectively, in the validation set used for the criteria [5, 8, 9]. When these arms were combined, the overall sensitivity and specificity became 82.9% and 84.4%, respectively, thus ensuring the best combination for the final criteria for axSpA. One of the challenges of these criteria is that the clinical arm is not as specific as the imaging arm. Few would question the accuracy of the classification in patients with sacroiliitis and/or MRI inflammation. However, the clinical arm is potentially much more lenient. In theory, a patient with a positive HLA-B27 and a family history of SpA could be classified as axSpA with a finding of enthesitis or dactylitis, even without a history of inflammatory back pain. As will be discussed below, this has important implications for therapy, where the response to tumor necrosis factor (TNF) inhibitors in the absence of sacroiliitis is clearly associated with the presence of active inflammation, as identified by either MRI findings or an elevated C-reactive protein (CRP) level. While the ASAS criteria allow for a balance between sensitivity and specificity, some might argue that specificity is the more critical issue when using this classification to consider the use of expensive therapies.

## Epidemiology

It has been difficult to determine the exact incidence and prevalence of axSpA in the US. Due to changing criteria and differing recognition of disease not specifically associated with radiographic findings, such as nr-axSpA, diagnostic delay and misdiagnosis can be very common. This is illustrated with differing prevalence data that have been reported in the US. According to the National Health and Nutrition Examination Survey (NHANES), approximately 1.7 million individuals aged 20–69 years in the USA suffer from spondyloarthritis as defined by the Amor and ESSG criteria [10]. However, the SPEED study, one of the first systematic studies to estimate axSpA prevalence using the ASAS criteria, estimated that 0.7% of the population aged 18–44 years is affected, or just under one million individuals [11]. Of these individuals, nr-axSpA and AS each accounted for about half of all axSpA cases in this study.

## AS versus nr-axSpA

The inflammation and eventual progression of joint damage in some individuals with axSpA can cause significant impairment in physical function [1]. Both AS and nr-axSpA are frequently associated with both peripheral articular and extra-articular disease; these are equally prevalent in both groups, with the exception of uveitis [12]. Uveitis appears to be more prevalent among patients with AS than nr-axSpA, a difference that is not clearly related to differences in HLA-B27 status [12]. Aside from uveitis, the clinical distinction between the two entities is not very pronounced, although nr-axSpA patients are more likely to be female [13, 14] and less likely to be HLA-B27^+^ [15]. Several studies have shown that patients with AS and nr-axSpA have similar levels of clinical disease as measured by the Bath Ankylosing Spondyloarthritis Disease Activity Index (BASDAI), functional status as measured by the Bath Ankylosing Spondyloarthritis Functional Index (BASFI), and health-related quality of life (HRQOL) measurements [13, 16, 17]. However, it can be argued that AS and nr-axSpA do differ slightly when considering nonclinical factors such as CRP levels and inflammation seen on MRI. In a trial that followed patients with AS and nr-axSpA during the first 2 years from diagnosis, CRP was higher in the AS group than in the nr-axSpA group, potentially indicating a burden of inflammation that was higher in patients with AS [16]. In a cohort of AS and nr-axSpA patients not treated with TNF inhibitors, AS patients with higher CRP levels did not spontaneously achieve low disease activity status as compared with their nr-axSpA counterparts [16]. This higher burden of inflammation is further supported by the finding of higher levels of inflammation seen on MRI in patients with AS [13, 18]. Despite the increased inflammation often seen in AS, response to TNF blockade has been similar between the two groups when inflammation is present [2, 17, 19], further supporting the fact that the two diagnoses are clinically comparable, and some have argued that the distinction may not be relevant when it comes to therapy [20]. Some studies have suggested that higher CRP levels and a higher degree of inflammation seen on MRI may play a role in the degree of treatment response for both AS and nr-axSpA, which could have relevance in AS if signs of inflammation are more prominent [17]. Finally, longer disease duration has also been associated with a divergence between the two entities. In the German Spondyloarthritis Inception Cohort (GESPIC) of patients with AS and nr-axSpA, AS patients (who had longer symptom duration at baseline) had significantly worse functional scores, and this difference was maintained over 2 years of follow-up [16].

## Disease monitoring

The BASDAI is a validated instrument for monitoring disease status/activity in patients with AS by assessing fatigue, pain (both axial and peripheral), morning stiffness, and tenderness (Table 1) [21]. While not validated in the broader diagnosis of axSpA, the ASAS group has suggested that BASDAI may be used to assess disease activity in patients across the spectrum of axSpA, including patients with nr-axSpA [3]. Another useful monitoring tool is the BASFI, which measures functional status and the ability to complete activities of daily living (Table 2) [22]. Both tools rely on patient questionnaires and are considered to be purely subjective and patient-oriented [23]. Clinical trials have typically relied on the ASAS response criteria, which measures improvement in four domains of patient-reported outcomes: global assessment, spinal pain, function, and inflammation. The BASDAI has been criticized for redundancy in its variables, assessment of only certain aspects of disease activity, and lack of specificity for inflammatory processes [23]. The ASAS response also lacks an objective measure of inflammation. The Ankylosing Spondylitis Disease Activity Score (ASDAS) was created to address these issues, most notably incorporating objective evidence of systemic inflammation in the form of a CRP level and including specific questions on axial and peripheral pain and stiffness drawn from the BASDAI. It has been shown to perform very well as a monitoring tool and is now widely considered superior to the BASDAI [23].

**Table 1:** Items in the Bath Ankylosing Spondylitis Disease Activity Index (BASDAI), scored on a numerical rating scale [17]

How would you describe the overall level of fatigue/tiredness you have experienced?	
How would you describe the overall level of ankylosing spondylitis neck, back or hip pain you have had?	
How would you describe the overall level of pain/swelling in joints other than neck, back or hips you have had?	
How would you describe the overall level of discomfort you have had from any areas tender to touch or pressure?	
How would you describe the overall level of morning stiffness you have had from the time you wake up?	
How long does your morning stiffness last from the time you wake up?

**Table 2:** Items in the Bath Ankylosing Spondylitis Functional Index (BASFI), scored on a numerical rating scale [18]

Putting on your socks or tights without help or aids (e.g., sock aid).	
Bending forward from the waist to pick up a pen from the floor without an aid.	
Reaching up to a high shelf without help or aids (e.g., helping hand).	
Getting up out of an armless dining room chair without using your hands or any other help	
Getting up off the floor without help from lying on your back.	
Standing unsupported for 10 min without discomfort.	
Climbing 12 to 15 steps without using a handrail or walking aid. One foot at each step.	
Looking over your shoulder without turning your body.	
Doing physically demanding activities (e.g., physiotherapy, exercises, gardening or sports).	
Doing a full days activities, whether it be at home or at work.	

## CRP

The biomarker of greatest interest in axial spondyloarthritis has been CRP, although its use and measurement is not without controversy. It general, patients with nr-axSpA have lower levels of CRP than similar patients with AS [13, 16], and this difference remains consistent even after receiving treatment [2, 16]. Despite this finding, some believe that CRP is not sensitive in axSpA [13] and overall has poor correlation with disease activity in both entities [18]. However, CRP has been shown to be a useful in its prognostic value with respect to progression and response to treatment. In studies that followed patients with axSpA over time (including both radiographic and nonradiographic disease), CRP positivity was the strongest risk factor associated with radiographic progression [18, 22, 24, 25]. A number of studies have shown an increased response to therapy, both with nonsteroidal anti-inflammatory drugs (NSAIDs) and TNF inhibitors, when baseline CRP or high-sensitivity CRP (hsCRP) was elevated, for both r-axSpA and nr-axSpA [2, 26, 27]. These findings seem to support the notion that objective findings of increased inflammation at baseline can predict response to TNF inhibitor therapy, which will be discussed in more detail below.

## Progression

While nr-axSpA and AS may fall on the same disease spectrum, not all patients with nr-axSpA will develop radiographic sacroiliitis, supporting the notion that nr-axSpA is not simply “early” AS [3, 5]. Additionally, not all patients with radiographic sacroiliitis will develop syndesmophytes [3]. Several observational studies have cited rates of progression from 10–12% of the population within the first 2 years [16, 18], to 20–25% 2–8 years after diagnosis [15], to 26–28% of patients after 10 or more years of observation [28, 29]. More recently, however, a prospective longitudinal study found that rates of progression actually may be lower than previously described. Dougados et al. followed patients from the Devenir des Spondylarthropathies Indifférenciées Récentes (DESIR) cohort for 5 years to evaluate progression of nr-axSpA to AS, and they found that only 5.1% had progressed [30]. Within this cohort, there were actually a few patients who “regressed” from AS to nr-axSpA [30]; as radiographic sacroiliitis should not reverse, this highlights the potential for variability in the interpretation of radiographs.

There are specific characteristics that have been identified as being associated with increased progression from nr-axSpA to AS. In a study comparing the rates of progression in nr-axSpA patients meeting the two diagnostic arms, subjects in the imaging arms were 3.5 times more likely to have progression to AS than those in the clinical arm [29]; elevation of CRP, like MRI that changes an indication of active inflammation, has also been associated with progression [18]. While cohort studies have not found clinical differences between the two arms in terms of disease severity, patients in the imaging arm were more likely to be male, younger, have a higher CRP level, and have a longer disease duration [31]. These risk factors were also demonstrated in the DESIR cohort longitudinal study described above, where bone marrow edema, longer symptom duration, and younger age were predictive of radiographic progression [30].

Besides establishing factors that are associated with the progression of nr-axSpA to AS, it is also important to note the progression of radiographic change within each entity itself. Poddubnyy et al. assessed an early disease patient cohort (GESPIC) that included both AS and nr-axSpA to determine progression and associated risk factors over a period of 2 years. They found that the strongest predictor of progression of radiographic sacroiliitis by at least one grade was an elevated CRP at baseline, which carried an associated increased risk of 3.65 [18]. Males and those with a longer duration of symptoms also had greater progression of sacroiliitis among the AS group, but not among the nr-axSpA group [18].

## Gender differences

Among patients with axSpA, gender differences have been identified in the distribution of disease along the spectrum. In general, proportionally more women have nr-axSpA than AS [13] and these women have a lower grade of inflammation seen on MRI [13, 31]. Even among patients with longstanding AS, women have been shown to have less radiographic damage than men [32, 33], as well as slower progression. Women also tend to have a stronger family history for both AS and early axial SpA [31, 33].

A multicenter prospective study (DESIR cohort) that specifically evaluated gender differences in patients with early axSpA found that extra-articular symptoms such as uveitis, enthesitis, psoriasis, and inflammatory bowel disease are more frequent in women [31]. Clinical disease, as measured by the BASDAI and functional scores, was worse in women; however, the ASDAS-CRP, which includes CRP and excludes peripheral symptoms, did not differ [31]. Given the higher degree of extra-articular disease and overall decreased functional status, it is possible that there may be an overdiagnosis of axSpA in women when using the clinical arm of the ASAS criteria [31]. Alternatively, underdiagnosis and undertreatment may also be possible in light of the sometimes atypical presentation in women [34] or the attribution of inflammatory pain to causes other than axSpA [1, 34], such as fibromyalgia. Both scenarios can pose a diagnostic dilemma for women with axSpA, and it may be helpful to be more liberal in the use of imaging to confirm the diagnosis in women with symptoms of inflammatory back pain [31].

There may also be gender differences in the progression of disease. While men with AS have greater rates of radiographic progression than women, men were shown to be have a lower probability of radiographic progression in one small study of sacroiliitis in nr-axSpA [18]. The impact of gender on progression of disease among nr-axSpA patients remains an area of investigation.

Finally, gender may affect response to treatment. In one study, women with nr-axSpA had a lower response rate to TNF inhibition compared to women with AS, a difference that was not seen in men [35]. While this study excluded patients with self-reported fibromyalgia, it is still possible that fibromyalgia or other noninflammatory conditions could be playing a role in clinical outcome measures. Fibromyalgia is considered to be the most common cause of generalized musculoskeletal pain in women aged 20–55 years [36]. In several studies involving patients with axSpA, fibromyalgia has been found to be a highly prevalent and a possible confounder for the diagnosis and assessment of axSpA [32, 33]. Salaffi et al. found that fibromyalgia was present in almost 15% of patients with axSpA, with a higher prevalence among women [37]. Disease monitoring for women with possible concurrent fibromyalgia and axSpA may be a challenge as well. BASDAI scores, which rely strictly on patient-reported outcomes, have been found to be higher in women with primary fibromyalgia than in those with AS [38], which may suggest an important role for ASDAS, rather than BASDAI, for disease monitoring in women with axSpA [37].

## Treatments

The mainstays of treatment for axSpA include both nonpharmacologic approaches and pharmacologic options. Nonpharmacologic approaches are largely based on physical therapy and exercise to maintain flexibility and normal posture [39] Additionally, lifestyle changes such as smoking cessation may of be some utility due to its association with disease activity and radiographic progression, possibly mediated through elevated CRP levels [40]. Furthermore, it has been shown that current smoking can impair response to TNF inhibition [41]. These nonpharmacologic interventions can be very useful in patients with axSpA, and they should be used in conjunction with pharmacologic treatments.

## NSAIDS and other oral agents

NSAIDs are considered the initial preferred therapy for all patients with symptomatic axSpA, including patients with both nonradiographic disease and AS [3, 39]. Current recommendations are to titrate up to the maximum dose and use these agents on a continuous basis, while taking into account tolerability and risk/benefit profile [3]. Interestingly, an extensive Cochrane review did not find a dose-dependent benefit with NSAIDs [42]. Although some believe that tolerability, and potentially treatment adherence, may be an issue [43], this same Cochrane review found a lack of adverse effects in clinical trials [42], although this must be balanced against recent evidence of cardiovascular risk with even short-term use of NSAIDs [44]. In addition to its symptom-reducing properties, it has also been suggested that NSAIDs may have a disease-modifying effect through bone growth inhibition via prostaglandin inhibition [45]. The clinical trial evidence for this has not been as clear [46, 47], however, and may be limited by the ethical challenge of conducting the long-term placebo-controlled trials necessary to definitively demonstrate a structural effect. One observational study showed that high NSAID use retarding new bone formation was most pronounced in patients with baseline syndesmophytes and elevated CRP levels [25]. Given this finding, it is not surprising that this study also showed that the effect on radiographic change was less prominent in individuals with nr-axSpA [25].

Considering all of this, the latest update to the ASAS recommendations for treatment of axSpA, including nonradiographic disease, recommends a trial of at least 1 month of maximally dosed NSAIDs prior to initiation of a biologic [3], with the caveat that benefits should outweigh the risks for the specific patient. The same recommendations state that purely axial disease should not be treated with conventional disease-modifying antirheumatic drugs (DMARDs), although sulfasalazine may be considered in patients with peripheral arthritis [3]. Steroids are recommended for use only as local injections to sites of musculoskeletal inflammation, such as monoarticular synovitis or enthesitis.

## TNF inhibitors

When NSAIDs fail to control disease symptoms, the next step is the use of biologic DMARDs, such as TNF inhibitors. TNF inhibition has been the therapy of choice for patients with established AS. TNF inhibitor use has also been studied in nr-axSpA (Table 3). Prior to the development of the ASAS criteria, which identified patients with axSpA and nonradiographic disease, two trials were completed in this population. One investigated adalimumab for the treatment of patients with axial spondyloarthritis without radiographically defined sacroiliitis and a second studied infliximab for the treatment of MRI-determined early sacroiliitis. Both trials demonstrated a significant improvement in ASAS scores after 12 weeks of treatment [48, 49]. Following publication of the ASAS criteria, the ABILITY-1 study examined the efficacy of adalimumab in patients with axSpA who did not meet criteria for AS (i.e., nr-axSpA). It, too, found improvements in ASDAS, BASDAI, functional scores, and radiographic inflammation in the spine and sacroiliac joints after 12 weeks of treatment compared to placebo [27]. Interestingly, the response to adalimumab in this trial was quite similar to the response to adalimumab in AS (Table 3). Of note, the response to adalimumab was greater in those patients with inflammatory changes on MRI and an elevated CRP; those with neither did not have significant improvement relative to placebo. Similarly, in the early sacroiliitis trial with infliximab, treatment and placebo ASAS40 rates were 61% and 18% at 24 weeks, compared with 47% and 12% in the pivotal ASSERT trial of infliximab in AS [50].

**Table 3:** Phase 3 trials of tumor necrosis factor (TNF) inhibitors in nonradiographic spondyloarthritis and ankylosing spondylitis

Trial	TNF inhibitor	Dose	Primary endpoint	Comparator	Results
nr-axSpA Trials					
ABILITY-1 [27]	Adalimumab	40 mg q 2 weeks	ASAS40 response at week 12	Placebo	ASAS20: 52% vs. 41% ASAS40: 36% vs. 15%
RAPID-axSpA (nr-axSpA subset) [19]	Certolizumab pegol	400 mg q 4 weeks	ASAS20 response at week 12	Placebo	ASAS20: 63% vs. 40% ASAS40: 47% vs. 16%
EMBARK [51]	Etanercept	50 mg q week	ASAS40 response at week 12	Placebo	ASAS20: 52% vs. 36% ASAS40: 33% vs. 15%
GO-AHEAD [26]	Golimumab	50 mg q 4 weeks	ASAS20 response at week 16	Placebo	ASAS20: 72% vs. 40% ASAS40: 57% vs. 23%
AS Trials					
ATLAS [56]	Adalimumab	40 mg q 2 weeks	ASAS20 response at week 12	Placebo	ASAS20: 58% vs. 21% ASAS40: 40% vs. 13%
RAPID-axSpA (AS subset) [19]	Certolizumab pegol	400 mg q 4 weeks	ASAS20 response at week 12	Placebo	ASAS20: 64% vs. 37% ASAS40: 50% vs. 19%
Etanercept phase III [57]	Etanercept	50 mg q week	ASAS20 response at week 12	Placebo	ASAS20: 59% vs. 28% ASAS50: 44% vs. 16%
GO-RAISE [53]	Golimumab	50 mg q 4 weeks	ASAS20 response at week 14	Placebo	ASAS20: 59% vs. 22% ASAS40: 44% vs. 15%

Etanercept has been evaluated for efficacy in the EMBARK study in nr-axSpA. In this study of early (<5 years of symptoms) nr-axSpA patients, Dougados et al. demonstrated benefit with etanercept compared to placebo as early as 2 weeks, with further improvement at 8 and 12 weeks [51]. As with adalimumab, the response to etanercept in the EMBARK study in nr-AxSpA was similar to that seen in the pivotal phase 3 trial in AS (Table 3). In the ESTHER trial, which included patients with both AS and nr-axSpA, etanercept was found to be superior to sulfasalazine in the reduction of inflammation seen on MRI in the spine, sacroiliac joints, and entheseal sites [52]. The clinical response rate for the two groups was similar and for some outcomes even superior for nr-axSpA [17].

Golimumab has also been studied in both AS and nr-axSpA and, again, the response was fairly comparable (Table 3). As with adalimumab, the ASAS40 response rates for golimumab in nrAxSpa were higher for those patients with an elevated CRP and inflammatory changes on MRI. At 14 weeks, the ASAS20 response rate for golimumab and placebo was 59%/22%, and the ASAS40 response rate was 44%/15% [53].

Finally, certolizumab has been evaluated in a clinical trial that included both AS and nr-axSpA patients in the same study [19]. In this study, which enrolled 325 patients meeting ASAS criteria for axSpA, 178 also met NY criteria for AS, while 147 were considered nr-axSpA, although all patients were required to have either MRI evidence of inflammation or an elevated CRP at study entry. This trial included two dosing regimens for certolizumab, 200 mg every 2 weeks and 400 mg every 4 weeks. At week 12, the ASAS20 response rates for certolizumab 400 mg every 4 weeks and placebo were 64% and 38%, and the ASAS40 response rates were 49% and 18%. When the results were analyzed by disease criteria, the ASAS20 and ASAS40 response rates for AS were 64% and 50%, and those for nr-axSpA were 63% and 47%.

Data such as these have led to the ASAS recommendation to extend TNF inhibitor treatment to the nr-axSpA subpopulation [19], although this recommendation is not yet accepted by all regulatory agencies worldwide. In Europe, for example, TNF inhibitors are indicated for axSpA, including nonradiographic disease, as long as there is evidence of active inflammation, while in the USA the indication is currently restricted to AS. As noted, several trials have shown that patients with a shorter disease duration (<5 years), elevated CRP, or sacroiliac joint inflammation seen on MRI may have a better response to therapy. Some trials have shown that symptom control remains equivalent between patients with nr-axSpA and AS on TNF inhibitor therapy at the 1-year follow-up [51] and even at a 3-year follow-up [2]. Despite the improvement in symptom and inflammation control, it must be noted that there is no firm evidence that TNF inhibitors are actually disease-modifying for axSpA [3]. This is certainly a relevant concern in AS; it is perhaps somewhat less relevant in nr-axSpA, where many patients may never develop progressive structural changes, and changes may be small in those who do, so that it would be difficult to prove meaningful benefit in this setting even with an agent believed to be truly disease-modifying.

The only other biologic currently approved for the treatment of AS is secukinumab, a monoclonal antibody directed against interleukin (IL)-17A [54]. Trials have shown the drug to be symptomatically effective compared with placebo [55]. Secukinumab has not been studied in nr-axSpA, nor are there any data available on its comparative efficacy relative to TNF inhibitors [54]. At this time, the ASAS recommendations are to treat with a TNF inhibitor after failure of two NSAIDs and to use it for at least 12 weeks [3]. In the event of an inadequate response, switching to another TNF inhibitor or treatment with an IL-17 inhibitor may be considered [3].

## Conclusion

The classification of spondyloarthritis remains an evolving topic. While there is general agreement on the current ASAS classification of axSpA, there is remaining uncertainty on whether clinical differences among patients meeting these criteria may have relevance for prognosis, disease progression, and response to therapy. Nonradiographic axSpA has emerged as one subclassification, though it appears to be part of the spectrum of axSpA rather than an entity in itself. As such, it currently seems to have more relevance as a regulatory construct than a disease definition. Other aspects of disease, such as gender, duration, and inflammatory burden, some of which may differ in those patients with nr-axSpA, appear to be more relevant to clinical course and treatment response. As these other demographic elements are better understood, particularly with respect to their impact on therapy, the concept of nr-axSpA will almost certainly become less important.

## Abbreviations

AS: Ankylosing spondylitis
ASAS: Assessment of SpondyloArthritis International Society
ASDAS: Ankylosing Spondylitis Disease Activity Score
axSpA: Axial spondyloarthritis
BASDAI: Bath Ankylosing Spondylitis Disease Activity Index
BASFI: Bath Ankylosing Spondylitis Functional Index
CRP: C-reactive protein
DESIR: Devenir des Spondylarthropathies Indifférenciées Récentes
DMARD: Disease-modifying antireheumatic drug
ESSG: European Spondyloarthropathy Study Group
GESPIC: German Spondyloarthritis Inception Cohort
HLA: Human leukocyte antigen
HRQOL: Health-related quality of life
hsCRP: High-sensitivity C-reactive protein
IL: Interleukin
mNY: Modified New York
MRI: Magnetic resonance imaging
NHANES: National Health and Nutrition Examination Survey
nr-axSpA: Nonradiographic axial spondyloarthritis
NSAID: Nonsteroidal anti-inflammatory drug
r-axSpA: Radiographic axial spondyloarthritis
SpA: Spondyloarthritis
TNF: Tumor necrosis factor

## References

[1] Strand V, Singh JA. Evaluation and management of the patient with suspected inflammatory spine disease. Mayo Clinic Proceedings. 2017;92(4):555–64. 10.1016/j.mayocp.2016.12.008 28233529

[2] Wallman JK (2015). Comparison of non-radiographic axial spondyloarthritis and ankylosing spondylitis patients—baseline characteristics, treatment adherence, and development of clinical variables during three years of anti-TNF therapy in clinical practice. Arthritis Res Ther.

[3] van der Heijde D (2017). 2016 update of the ASAS-EULAR management recommendations for axial spondyloarthritis. Ann Rheum Dis..

[4] Linden SVD, Valkenburg HA, Cats A (1984). Evaluation of diagnostic criteria for ankylosing spondylitis. Arthritis Rheumatol.

[5] Sieper J, van der Heijde D (2013). Review: nonradiographic axial spondyloarthritis: new definition of an old disease?. Arthritis Rheum.

[6] Lipton S, Deodhar A (2012). The new ASAS classification criteria for axial and peripheral spondyloarthritis: promises and pitfalls. Int J Clin Rheumatol.

[7] Taylor WJ, Robinson PC (2013). Classification criteria: peripheral spondyloarthropathy and psoriatic arthritis. Curr Rheumatol Rep.

[8] Rudwaleit M (2009). The development of Assessment of SpondyloArthritis international Society classification criteria for axial spondyloarthritis (part I): classification of paper patients by expert opinion including uncertainty appraisal. Ann Rheum Dis.

[9] Rudwaleit M, Landewe R, van der Heijde D (2009). SpondyloArthritis international Society (ASAS) classification criteria for axial spondyloarthritis (part II): validation and final selection. Ann Rheum Dis.

[10] Reveille JD, Witter JP, Weisman MH (2012). Prevalence of axial spondylarthritis in the United States: estimates from a cross‐sectional survey. Arthritis Care Res.

[11] Strand V (2013). Prevalence of axial spondyloarthritis in United States rheumatology practices: Assessment of SpondyloArthritis International Society criteria versus rheumatology expert clinical diagnosis. Arthritis Care Res.

[12] de Winter JJ (2016). Prevalence of peripheral and extra-articular disease in ankylosing spondylitis versus non-radiographic axial spondyloarthritis: a meta-analysis. Arthritis Res Ther.

[13] Kiltz U (2012). Do patients with non‐radiographic axial spondylarthritis differ from patients with ankylosing spondylitis?. Arthritis Care Res.

[14] Rudwaleit M (2009). The early disease stage in axial spondylarthritis: results from the German Spondyloarthritis Inception Cohort. Arthritis Rheum.

[15] Sampaio-Barros PD (2010). Undifferentiated spondyloarthritis: a long-term follow-up. J Rheumatol.

[16] Poddubnyy D (2015). Brief report: clinical course over two years in patients with early nonradiographic axial spondyloarthritis and patients with ankylosing spondylitis not treated with tumor necrosis factor blockers: results from the German Spondyloarthritis Inception Cohort. Arthritis Rheumatol.

[17] Song I-H (2013). Similar response rates in patients with ankylosing spondylitis and non-radiographic axial spondyloarthritis after 1 year of treatment with etanercept: results from the ESTHER trial. Ann Rheum Dis.

[18] Poddubnyy D (2011). Rates and predictors of radiographic sacroiliitis progression over 2 years in patients with axial spondyloarthritis. Ann Rheum Dis.

[19] Landewe R (2014). Efficacy of certolizumab pegol on signs and symptoms of axial spondyloarthritis including ankylosing spondylitis: 24-week results of a double-blind randomised placebo-controlled phase 3 study. Ann Rheum Dis..

[20] Deodhar A (2016). The term ‘non-radiographic axial spondyloarthritis’ is much more important to classify than to diagnose patients with axial spondyloarthritis. Ann Rheum Dis..

[21] Garrett S (1994). A new approach to defining disease status in ankylosing spondylitis: the Bath Ankylosing Spondylitis Disease Activity Index. J Rheumatol.

[22] Calin A (1994). A new approach to defining functional ability in ankylosing spondylitis: the development of the Bath Ankylosing Spondylitis Functional Index. J Rheumatol.

[23] Machado P, van der Heijde D (2011). How to measure disease activity in axial spondyloarthritis?. Curr Opin Rheumatol.

[24] Poddubnyy D (2012). Baseline radiographic damage, elevated acute‐phase reactant levels, and cigarette smoking status predict spinal radiographic progression in early axial spondylarthritis. Arthritis Rheum.

[25] Poddubnyy D (2012). Effect of non-steroidal anti-inflammatory drugs on radiographic spinal progression in patients with axial spondyloarthritis: results from the German Spondyloarthritis Inception Cohort. Ann Rheum Dis.

[26] Sieper J (2015). A randomized, double‐blind, placebo‐controlled, sixteen‐week study of subcutaneous golimumab in patients with active nonradiographic axial spondyloarthritis. Arthritis Rheumatol.

[27] Sieper J (2013). Efficacy and safety of adalimumab in patients with non-radiographic axial spondyloarthritis: results of a randomised placebo-controlled trial (ABILITY-1). Ann Rheum Dis.

[28] Ruderman E (2013). Spondyloarthritis Epidemiology and Burden Phase 2 [speed 2] Study: Disease progression in axial spondyloarthropathy (SpA). Arthritis Rheum..

[29] Wang R, Gabriel SE, Ward MM (2016). Progression of nonradiographic axial spondyloarthritis to ankylosing spondylitis: a population‐based cohort study. Arthritis Rheumatol.

[30] Dougados M, Sepriano A, Molto A, Ramiro S, de Hooge M, Van Den Berg R, Navarro Compan V, De Mattei C, Landewé R, Van der Heijde D. Evaluation of the changes in structural damage in axial spondyloarthritis on plain pelvic radiographs: the 5 years data of the DESIR cohort. Ann Rheum Dis. 2017;76(supplement 2):100. 10.1136/annrheumdis-2017-211596 PMC5705846 28684556

[31] Tournadre A (2013). Differences between women and men with recent‐onset axial spondyloarthritis: results from a prospective multicenter french cohort. Arthritis Care Res.

[32] Aloush V (2007). Fibromyalgia in women with ankylosing spondylitis. Rheumatol Int.

[33] Lee W (2007). Are there gender differences in severity of ankylosing spondylitis? Results from the PSOAS cohort. Ann Rheum Dis.

[34] Slobodin G (2011). Recently diagnosed axial spondyloarthritis: gender differences and factors related to delay in diagnosis. Clin Rheumatol.

[35] Ciurea AHM, Weber U, Tamborrini G, Micheroli R, Wildi L, Zufferey P, Nissen MJ, Villiger PM, Bernhard J, van der Heijde D, Landewé RBM, Scherer A, Exer P. In contrast to men, women with nonradiographic axial spondyloarthritis have lower response rates to TNF inhibitors than women with ankylosing spondylitis. ACR. 2016;2016:Abstract 699.

[36] Atzeni F (2011). Chronic widespread pain in the spectrum of rheumatological diseases. Best Pract Res Clin Rheumatol.

[37] Salaffi F (2014). Fibromyalgia in patients with axial spondyloarthritis: epidemiological profile and effect on measures of disease activity. Rheumatol Int.

[38] Heikkilä S (2002). Functional impairment in spondyloarthropathy and fibromyalgia. J Rheumatol.

[39] Ward MM (2016). American College of Rheumatology/Spondylitis Association of America/Spondyloarthritis Research and Treatment Network 2015 recommendations for the treatment of ankylosing spondylitis and nonradiographic axial spondyloarthritis. Arthritis Rheumatol.

[40] Poddubnyy D (2013). Cigarette smoking has a dose-dependent impact on progression of structural damage in the spine in patients with axial spondyloarthritis: results from the GErman SPondyloarthritis Inception Cohort (GESPIC). Ann Rheum Dis.

[41] Ciurea A (2016). Impaired response to treatment with tumour necrosis factor α inhibitors in smokers with axial spondyloarthritis. Ann Rheum Dis..

[42] Kroon FP (2016). Nonsteroidal antiinflammatory drugs for axial spondyloarthritis: a Cochrane review. J Rheumatol.

[43] Varkas G (2015). OP0170 treatment of axial spondyloarthritis with an optimal dosage of nsaids: a 6-week follow up study focusing on magnetic resonance imaging of the sacroiliac joints. Ann Rheum Dis.

[44] Bally M (2017). Risk of acute myocardial infarction with NSAIDs in real world use: Bayesian meta-analysis of individual patient data. BMJ..

[45] Blackwell KA, Raisz LG, Pilbeam CC (2010). Prostaglandins in bone: bad cop, good cop?. Trends Endocrinol Metab.

[46] Sieper J (2008). Comparison of two different dosages of celecoxib with diclofenac for the treatment of active ankylosing spondylitis: results of a 12-week randomised, double-blind, controlled study. Ann Rheum Dis.

[47] Wanders A (2005). Nonsteroidal antiinflammatory drugs reduce radiographic progression in patients with ankylosing spondylitis: a randomized clinical trial. Arthritis Rheum.

[48] Barkham N (2009). Clinical and imaging efficacy of infliximab in HLA-B27-positive patients with magnetic resonance imaging-determined early sacroiliitis. Arthritis Rheumatol.

[49] Haibel H (2008). Efficacy of adalimumab in the treatment of axial spondylarthritis without radiographically defined sacroiliitis: results of a twelve‐week randomized, double‐blind, placebo‐controlled trial followed by an open‐label extension up to week fifty‐two. Arthritis Rheum.

[50] van der Heijde D (2005). Efficacy and safety of infliximab in patients with ankylosing spondylitis: results of a randomized, placebo‐controlled trial (ASSERT). Arthritis Rheumatol.

[51] Dougados M (2014). Symptomatic efficacy of etanercept and its effects on objective signs of inflammation in early nonradiographic axial spondyloarthritis: a multicenter, randomized, double‐blind, placebo‐controlled trial. Arthritis Rheumatol.

[52] Song I (2011). Effects of etanercept versus sulfasalazine in early axial spondyloarthritis on active inflammatory lesions as detected by whole-body MRI (ESTHER): a 48-week randomised controlled trial. Ann Rheum Dis.

[53] Inman RD (2008). Efficacy and safety of golimumab in patients with ankylosing spondylitis: results of a randomized, double-blind, placebo-controlled, phase III trial. Arthritis Rheum.

[54] Cheung PP (2017). Anti-IL17A in axial spondyloarthritis—where are we at?. Front Med..

[55] Baeten D (2015). Secukinumab, an interleukin-17A inhibitor, in ankylosing spondylitis. N Engl J Med.

[56] van der Heijde D (2006). Efficacy and safety of adalimumab in patients with ankylosing spondylitis: results of a multicenter, randomized, double-blind, placebo-controlled trial. Arthritis Rheum.

[57] Davis JC (2003). Recombinant human tumor necrosis factor receptor (etanercept) for treating ankylosing spondylitis: a randomized, controlled trial. Arthritis Rheumatol.

